# Pituitary gigantism: a case series from Hospital de San José (Bogotá, Colombia)

**DOI:** 10.20945/2359-3997000000150

**Published:** 2019-07-11

**Authors:** William Rojas García, Henry Tovar Cortes, Andrés Florez Romero

**Affiliations:** 1 Hospital de San José Endocrinology Unit Hospital de San José Colombia Head of the Endocrinology Unit, Hospital de San José;; Fundación Universitaria de Ciencias de la Salud Bogotá DC Colombia associate professor, Fundación Universitaria de Ciencias de la Salud, Bogotá, DC, Colombia; 2 Hospital de San José Colombia Hospital de San José;; Fundación Universitaria de Ciencias de la Salud Bogotá DC Colombia assistant professor, Fundación Universitaria de Ciencias de la Salud, Bogotá, DC, Colombia; 3 Hospital de San José Bogotá DC Colombia Hospital de San José, Bogotá, DC, Colombia

**Keywords:** Pituitary diseases, gigantism, growth hormone, pituitary neoplasms, acromegaly

## Abstract

**Introduction:**

Gigantism is a rare pediatric disease characterized by increased production of growth hormone (GH) before epiphyseal closure, that manifests clinically as tall stature, musculoskeletal abnormalities, and multiple comorbidities.

**Materials and methods:**

Case series of 6 male patients with gigantism evaluated at the Endocrinology Service of Hospital de San José (Bogotá, Colombia) between 2010 and 2016.

**Results:**

All patients had macroadenomas and their mean final height was 2.01 m. The mean age at diagnosis was 16 years, and the most common symptoms were headache (66%) and hyperhidrosis (66%). All patients had acral changes, and one had visual impairment secondary to compression of the optic chiasm. All patients underwent surgery, and 5 (83%) required additional therapy for biochemical control, including radiotherapy (n = 4, 66%), somatostatin analogues (n = 5, 83%), cabergoline (n = 3, 50%), and pegvisomant (n = 2, 33%). Three patients (50%) achieved complete biochemical control, while 2 patients showed IGF-1 normalization with pegvisomant. Two patients were genetically related and presented a mutation in the aryl hydrocarbon receptor-interacting protein (AIP) gene (pathogenic variant, c.504G>A in exon 4, p.Trp168*), fulfilling the diagnostic criteria of familial isolated pituitary adenoma.

**Conclusions:**

This is the largest case series of patients with gigantism described to date in Colombia. Transsphenoidal surgery was the first-choice procedure, but additional pharmacological therapy was usually required. Mutations in the AIP gene should be considered in familial cases of GH-producing adenomas.

## INTRODUCTION

Gigantism is a rare pediatric disease, with an incidence of 8 to 11 cases per million individuals per year. This disease is characterized by increased production of growth hormone (GH) when the epiphyses are still open, and in most cases is secondary to a pituitary adenoma (1). Gigantism can occur sporadically or have a hereditary component (2); in a case series by Rostomyan and cols. (3), a genetic cause was identified in 46% of the cases, of which the most common was a mutation in the hydrocarbon receptor-interacting protein (*AIP*) gene (28%), followed by X-linked acrogigantism (X-LAG; 10%). McCune-Albright syndrome (5%), Carney complex (1%), and multiple endocrine neoplasia type 1 (1%) are less common causes of gigantism (3). The main symptom of the disease is abnormal accelerated growth affecting the musculoskeletal system associated with some other comorbidities (1). The first-choice treatment for gigantism is transsphenoidal surgery (TSS) (4). However, complete remission of the disease is not usually achieved with surgical intervention alone and pharmacological therapy becomes necessary (2,5-7), of which somatostatin analogues (SSA) is the most common. If no response is obtained with SSAs, dopamine receptor agonists (cabergoline) or GH receptor antagonists (pegvisomant) can be added (8-10). In cases that fail to respond to surgery and pharmacological treatment, radiotherapy is used; however, the risk of hypopituitarism should be taken into account (8).

The purpose of this study is to present 6 cases of gigantism treated in Colombia, including a 6-year follow-up and treatment outcomes. We also present the clinical history of 2 patients with gigantism secondary to familial isolated pituitary adenoma (FIPA) and *AIP* mutation.

## MATERIALS AND METHODS

We present a review of 6 cases of gigantism secondary to pituitary adenomas, managed at the Endocrinology Department of *Hospital de San José* (Bogotá, Colombia), a tertiary referral center, between January 2010 and December 2016. At this institution, we see an average of 110 cases of acromegaly per year. All patients provided a written informed consent for picture release. Data, including medical history and laboratory results, were collected retrospectively. Additional information was obtained directly from the patients.

*AIP* testing was requested from all patients but was only obtained from patients #1 and #5 (Table 1), confirming a diagnosis of FIPA with *AIP* mutation. Total genomic DNA extraction was performed from venous blood samples using conventional techniques, and an analysis of the complete *AIP* gene coding sequence (exons 1-6) was done including all exon-intron junctions. The exon sequences were compared against the GenBank accession number NM_003977.2, with the A of the ATG translation initiation codon in position 1. To test for the c.504G>A (p.Trp168*) variant of the *AIP* gene, total genomic DNA was extracted from venous blood samples following a conventional technique. A conventional PCR assay was developed to amplify exon 4 of the *AIP* gene (wild type sequence*,* ENST00000279146) from DNA in both cases. The amplified product was purified and sequenced.

**Table 1:** Demographic characteristics of patients with gigantism

Patient	Gender	Age at symptom onset (years)	Age at diagnosis (years)	Final height (meters)	Height - father (meters)	Height - mother (meters)	Z-score population mean	Z-score mean parental height	Body weight (kg)	BMI (kg/m^2^)	Tumor size (mm)	ST	RT	MED
1	M	12	12	1.96	1.71	NA	4.2	NA	107	27.8	5 x 17.3 x 29	TSS (2)^1^	YES	LAR, PEG
2	M	13	21	2.2	1.68	1.68	5.9	6.17	108	22.3	18 x 20 x 20	FC	NO	NA
3	M	11	11	2.1	1.58	1.5	4.5	6.69	103	23.3	NA	FC	YES	OCT, CAB
4	M	14	17	1.93	1.7	1.5	2.26	3.63	75	20.35	16 x 15 x 17	TSS	NO	OCT
5	M	14	23	1.91	NA	1.53	1.98	NA	122	33	NA	TSS	YES	NO
6	M	10	12	2	1.65	1.5	3.2	2.78	107	27.2	25 x 20 x 20	TSS (2)^1^	YES	LAR, PEG

CAB: Cabergoline, FC: Frontal craniotomy, LAR: Lanreotide, M: Male, MED: Medical treatment, NA: Not available, OCT: octreotide long-acting (LAR), PEG: Pegvisomant, ST: Surgical treatment, RT: Radiotherapy, TSS: Transsphenoidal surgery. 1. Number of surgeries performed.

The diagnosis of gigantism was established based on a height above 2 or more standard deviations for age (> 97th percentile), or a final height greater than 2 standard deviations above the general population, using the Colombian height and weight chart (11). Biochemical and imaging diagnostic tests included increased serum GH and insulin-like growth factor-1 (IGF-1) levels and evidence of a pituitary adenoma in the sella turcica on magnetic resonance imaging (MRI) (2,3). Since no standard criteria are available to define controlled disease in patients in gigantism, the biochemical diagnostic criteria for acromegaly were used for follow-up (12), *i.e.*, IGF-1 in the normal range and GH level below 1 ng/mL.

## RESULTS

In all, 6 cases of gigantism were managed at our Unit according to established criteria between 2010 and 2016. The patients were all male and had a mean age at symptom onset of 12.3 years. Their mean age at diagnosis was 16 years, and their mean final height was 2.01 meters (m). All patients had pituitary macroadenomas. The tumor sizes are described in Table 1. No record is available regarding the tumor sizes of patients #3 and #5 since they arrived at our center after undergoing surgical procedures at another institution, so initial MRI reports were not available. The most common symptoms were headache and hyperhidrosis, which were present in 4 patients, followed by acroparesthesia in 3 patients, and arthralgia and fatigue in 2 patients each.

All 6 patients showed acral changes. One patient (patient #1) had visual impairment secondary to compression of the optic chiasm by the adenoma. Only 2 patients (#1 and #5), had a family history of tall stature or other endocrine disorders. Table 1 presents a summary of the main clinical and laboratory findings of each patient.

All 6 patients were initially managed with surgical resection of the tumor, including TSS in 4 patients (#1, #4, #5, and #6) and frontal craniotomy in 2 patients (#2 and #3). Two patients required a second surgical intervention via TSS (patients #1 and #6). Immunohistochemistry confirmed exclusive production of GH by the adenomas in all patients, and none of the patients had increased serum prolactin.

All 6 patients had only partial improvement of symptoms after surgery and required other treatments. Four patients (#1, #3, #5, and #6) received radiotherapy, and 5 required additional medical management with SSAs (3 patients with lanreotide Autogel and 2 with long-acting release [LAR] octreotide). Due to the absence of clinical response, cabergoline was added to the therapy in 3 patients (#1, #3, #6) and pegvisomant was added to 2 patients (#1 and #6, both at a dose of 20 mg/day).

Normal GH (< 1 ng/mL) and IGF-1 levels were achieved in 4 patients, one after frontal craniotomy (patient #2); one after frontal craniotomy, radiotherapy, octreotide LAR, and cabergoline (patient #3); and 2 after TSS, lanreotide Autogel, pegvisomant, and radiotherapy (patients #1 and #6). One patient (#4), who received treatment with TSS and octreotide LAR, showed fluctuating IGF-1 levels, but since he was asymptomatic, pegvisomant was not recommended. Patient #5 interrupted the follow-up at our institution. Four patients (#1, #3, #4, and #6) underwent regular monitoring for more than 3 years, and their IGF-1 values are presented in Table 2. Patient #3 started following up at our center after undergoing surgical intervention, radiotherapy, and pharmacological treatment at another institution, therefore, his initial IGF-1 levels at our institution were normal.

**Table 2:** IGF-1 levels (in ng/mL) and upper limit of normal in patients followed up for more than 36 months

Patient	Months															

	0		6		12		18		24		30		36		42	

	ng/mL	ULN	ng/mL	ULN	ng/mL	ULN	ng/mL	ULN	ng/mL	ULN	ng/mL	ULN	ng/mL	ULN	ng/mL	ULN
1	794	1.6	824	1.6	1017	2	876	1.7	1084	2.1	692	1.4	700	1.4	130	N
3	61	N			85	N	39	N	26.2	N			25.9	N		
4	337	N	367	N	147	N	444	N	501	1.01	303	N	180	N	513	1.03
6	753	1.5	677	1.3	610	1.2	511	1.02	950	1.9	543	1.09	402	N	150	N

N: Normal, ULN: Upper limit of normal.

Regarding associated comorbidities, one patient (#5) had class 1 obesity, 2 (#1 and #6) were overweight, and one (#1) had hyperglycemia. Cholelithiasis was investigated with hepatobiliary ultrasound, but none of the patients presented this comorbidity. No other comorbidities associated with GH excess were found. Two patients presented hypopituitarism (patients #1 and #3, who had thyroid and gonadal dysfunctions, respectively).

**Figure 1:**
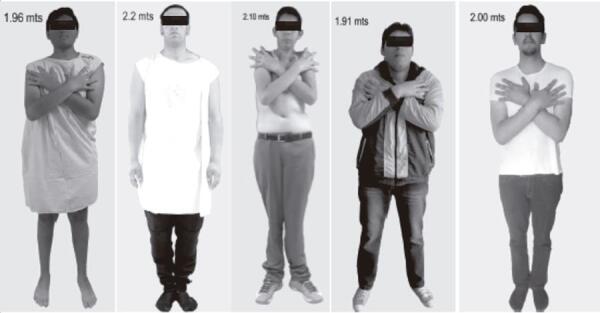
Photograph of 5 of the patients. From left to right: patient 1, patient 2, patient 3, patient 5 and patient 6.

**Figure 2:**
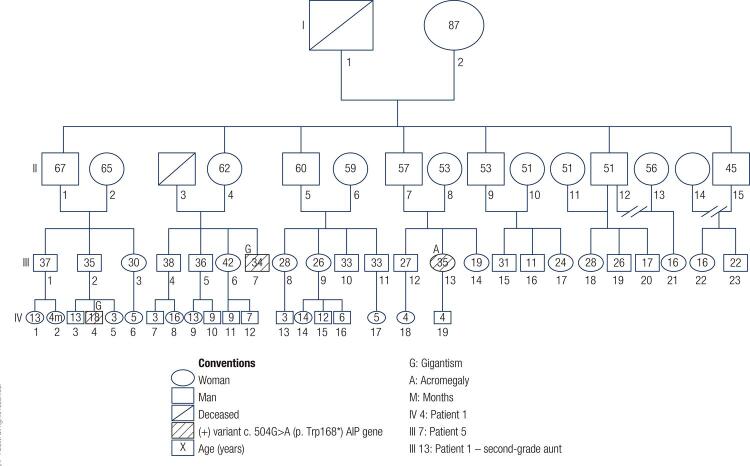
Genealogical family tree with mutation of the AIP gene.

Figure 1 shows pictures of 5 out of the 6 patients. A summary of the clinical history of the 2 patients (#1 and #5) who had familial pituitary adenomas is presented below. A genealogical tree of these patients (who belonged to the same family), from whom a sample of the *AIP* gene was requested, is presented in Figure 2.

**Figure 3:**
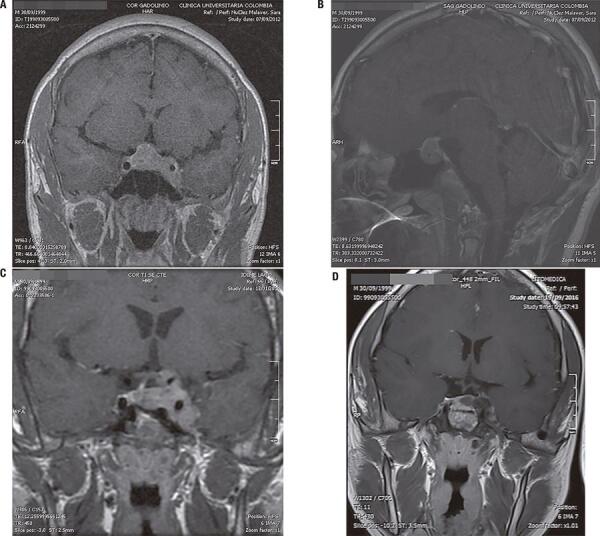
(A) Initial coronal T1-weighted magnetic resonance imaging (MRI). (B) Initial sagittal T1-weighted post-contrast MRI. (C) Coronal T1-weighted MRI 1 year after the second surgical procedure. (D) Coronal T1-weighted post-contrast MRI postcontrast 3 years after the second surgical procedure and 2 year after radioterapy plus medical treatment.

Patient 1: His symptoms began at the age of 12 years with headache, hyperhidrosis, lower limb paresthesia, and tall stature (1.74 m). His initial laboratory tests showed GH levels greater than 40 ng/mL (reference range 0-5 ng/mL), IGF-1 level 794 ng/mL (reference range 111-498 ng/mL), and normal levels of TSH, free T4, FSH, LH, prolactin, cortisol, ACTH, and glucose. An MRI of the sella turcica showed an expansive lesion of 5.0 x 17.3 x 29.0 mm compressing the optic chiasm and infiltrating the left cavernous sinus (Figures 3A and 3B). Monthly lanreotide (90 mg, intramuscular) was initiated and TSS was performed at the age of 12 years. Immunohistochemistry analysis of the adenoma was positive for GH. After surgery, the patient persisted with symptoms and acral growth and presented serum levels of GH > 40 ng/mL and IGF-1 of 1165 ng/mL; based on that, the lanreotide dose was increased to 120 mg and cabergoline 0.5 mg weekly was initiated. A new MRI showed a residual tumor infiltrating the left cavernous sinus, and a second TSS was performed at the age of 13 years. Due to poor biochemical control during postsurgical follow-up, his cabergoline dose was increased to 2 mg weekly. A follow-up MRI after the second surgery showed a lesion of 36 x 30 x 20 mm and optic chiasm compression (Figure 3C). Due to the increase in tumor size and poor biochemical control, radiotherapy was performed at the age of 14 years. Pegvisomant was also initiated and cabergoline was suspended, resulting in a decrease in IGF-1 levels and control of the symptoms. A contrast MRI performed 2 years after the radiotherapy is shown in Figure 3D. The final height of the patient was 1.96 m. Given the occurrence of gigantism in a second-degree uncle (patient #5) and acromegaly in a second-degree aunt, an *AIP* gene sequencing was requested, which showed the heterozygous pathogenic variant c.504G>A in exon 4 (p.Trp168*) generating a nonsense substitution of tryptophan causing a premature stop codon.

Patient 5: The onset of his symptoms occurred at the age of 14 years, manifesting as tall stature. The patient was diagnosed with a pituitary macroadenoma at the age of 23 years and treatment with TSS was performed. He required radiotherapy at the age of 27 years and used SSA for 1 year. Adequate biochemical and imaging control were observed at follow-up, and his final height was 1.91 m. Given the family history of gigantism on a second-degree nephew (patient #1) and acromegaly on a second-degree female cousin, the variant c.504G> A (p.Trp168*) of the *AIP* gene was tested and resulted positive. The same variant was tested in the patient’s relatives (patient #1 and patient #5, who was diagnosed with acromegaly at the age of 25 years), and resulted positive.

## DISCUSSION

Between 5-15% of the pediatric pituitary adenomas produce GH. Most cases (90%) comprise macroadenomas, and 30-60% are invasive. A higher frequency in males is reported in the literature (13). In most recent case series, there was a predominance of male patients, as in the series by Nagata and cols. (Japan; 7 out of 13 patients [54%]) (14), Creo and Lteif (USA; 9 out of 13 patients [69%]) (15), Rostomyan and cols. (Belgium; 163 out of 208 patients [78%]) (3), Patt and cols. (India; 13 out of 14 patients [92%]) (16), Mangupli and cols. (Venezuela) (6 out of 8 patients [75%]) (6), and in the present case series (Colombia; 6 out of 6 patients [100%]). The diagnosis of gigantism is usually established around the age of 14 years, and was reported at a mean age of 13.6 years by Creo and Lteif (15), 13 years by Rostomyan and cols. (3), 18 years by Mangupli and cols. (6), and 21.9 ± 6.1 years by Patt and cols. (16). In our case series, the diagnosis of gigantism was established at a mean age of 16 years. A delayed diagnosis of gigantism may occur due to poor perception of the magnitude of the symptoms, delayed consultations, and limited knowledge of the disease by healthcare providers, all of which are important factors in Latin America. The most common symptoms presented by our patients were headache (66%) and hyperhidrosis (66%), unlike the series by Rostomyan and cols. (3), in which headache was less frequent (23%).

In gigantism, TSS is the first-choice procedure, and biochemical control is obtained in 70% of the patients with intrasellar microadenomas, although this rate is lower with macroadenomas (13). TSS was the most common procedure performed in our patients (66%), and none of the patients obtained biochemical control with this treatment alone. The only case with biochemical control followed a frontal craniotomy. In the series by Nagata and cols*.*, 92% (12 out of 13) of the patients were managed with TSS and 53% (7 out of 13) achieved biochemical control; this was the case series with best reported response with TSS (14). In the study by Creo and Lteif, 92% (12 out of 13) of the patients were treated with TSS, and only 23% (3 out of 13) achieved biochemical control (15). In the publication by Rostomyan and cols., surgery was performed in 82% (177 out of 208) of the patients and only 15% obtained biochemical control (3). In the series by Patt and cols., surgery was performed in 92% (13 out of 14) of the patients and 21% obtained biochemical control (16). In the cases described by Mangupli and cols. (6), none of the patients who underwent surgery obtained control. In our series, surgical reintervention was required in 33% (2 out of 6) of the patients, compared with 30% (4 out of 13) in the series by Creo and Lteif (15) and 64% in the series by Rostomyan and cols. (3). Only 7.5% of our patients had a biochemical response to the surgical reintervention, while 2 patients (33%) presented hypopituitarism with thyroid and gonadal dysfunction, a rate that is similar to that reported by Creo and Lteif, who described a 38% rate of hypopituitarism (5 out of 13 patients) (15).

Given the low biochemical control with surgery, patients with gigantism usually require additional management. SSAs were used in 83% of our patients (5 out of 6; 3 were treated with lanreotide Autogel and 2 with octreotide LAR). This finding was similar in the series by Mangupli and cols. (6), in which 100% (8 out of 8) of the patients used SSAs. Still, the use of SSA was less frequent in other studies: 50% (6 out of 12) of the patients in the series by Creo and Lteif (15), 66.7% (118 out of 208) reported by Rostomyan and cols. (3), and 23% (3 out of 13) in the study by Nagata and cols. (14). In our series, none of the patients had biochemical control with SSA alone, which is aligned to the results by Creo and Lteif (15) and Mangupli and cols. (6); in contrast, in the study by Rostomyan and cols., 34% of the patients were controlled with SSA alone (3). Rates of biochemical control in patients with acromegaly have been reported at 63.9% with octreotide and 78.1% with lanreotide Autogel (17). Of note, one case report of a girl with gigantism and microadenoma showed biochemical control and disappearance of the tumor with octreotide LAR for 3 years (7). A prospective study conducted in Japan with 32 patients with acromegaly (29 patients) and gigantism (3 patients) assessed the efficacy of lanreotide Autogel; although separate data for patients with gigantism were not reported, the efficacy was reported to be similar in both groups (acromegaly and gigantism) (18).

Dopamine receptor agonists are useful in cases with associated hyperprolactinemia or as an adjunct therapy to SSAs in cases with lack of biochemical control and IGF-1 levels up to 1.5 times above the normal range (19). None of our patients presented hyperprolactinemia, in contrast to the finding by Mangupli and cols., in which 50% (4 out of 8) of the patients had hyperprolactinemia (6). Cabergoline was administered to 3 of our patients, and biochemical control was obtained in 1 (patient #3) with concomitant use of octreotide LAR. Cabergoline was administered to 4 patients by Mangupli and cols. (6) and 2 patients by Nagata and cols. (14), and none of the patients obtained biochemical control. The effectiveness of cabergoline in the management of gigantism without associated hyperprolactinemia lacks evidence.

Pegvisomant (a GH receptor antagonist) has been used in pediatrics to obtain IGF-1 normalization and symptom improvement in patients without a response to surgical treatment, radiotherapy, and SSAs, although the possibility of an increase in tumor size with this medication should be considered (9,13,20). In a report of 3 patients treated with pegvisomant, linear growth was interrupted after 6 months of treatment, and improvement in diaphoresis and facial features of acromegaly was observed, along with normalization of IGF-1 levels in 2 of them, while the other one showed an increase in tumor size (9). Effectiveness was confirmed in 2 of our patients in whom this medication was administrated (patients #1 and #6), a result that is similar to the one reported by Creo and Lteif (15) and Mangupli and cols. (6). A lower rate of biochemical control (50% of the patients) was observed by Rostomyan and cols. (3), and absence of response was observed on a single patient treated with pegvisomant by Nagata and cols. (14). The combination of SSAs with pegvisomant seems to be the most effective association for the treatment of gigantism, as reported by Mangupli and cols. in 8 patients with gigantism. Early (1 to 4 months) symptom control was observed, with an absence of tumor growth and normalization of IGF-1 levels in all patients (6).

Radiotherapy was administered to 66% (4 out of 6) of our patients, which is a higher rate than reported by other authors of case series: 46% (6 out of 13) of the patients by Creo and Lteif (15), 30% (63 out of 208) by Rostomyan and cols. (3), 35% (5 out of 14) by Patt and cols. (16), and one patient by Mangupli and cols. (6) and Nagata and cols. (14). The risk of hypopituitarism, which can occur in 30-50% of the patients, should be considered (13). Table 3 presents a comparison of case series of gigantism (3,6,14-16), including the present study. Most of the cases reported (78%) include men. The mean age at diagnosis was 15.1 years, and the mean final height was 1.95 m. TSS was the most frequent initial procedure (83%), and only 18% of the patients obtained biochemical control with this procedure alone, while 122 out of 219 patients (56%) required surgical reintervention. Half of the patients (53%) received SSAs, and only 1 successful case of treatment with SSA monotherapy was reported. Pegvisomant was administered to 17.5% of the patients, with IGF-1 normalization in 58% of them, which is a lower rate than the 67.5% response reported in a “real world” study in patients with acromegaly (21), possibly related to inadequate dose titration. Radiotherapy was used in 30% of the patients (Table 3), which is a high percentage taking into account the risk of hypopituitarism in this population.

**Table 3:** Comparison of case series of gigantism reported in the literature

	Rostomyan and Daly (multicentric) 2015	Patt and cols. (India) 2015	Creo and cols. (USA) 2016	Mangupli and cols. (Venezuela) 2016	Nagata and cols. (Japan) 2017	Rojas and cols. (Colombia) 2018 (current study)	Total
Male gender	163/208	14/14	9/13	6/8	7/13	6/6	205/262 (78%)
Mean final height (meters)	NA	1.87	2.05	1.9	NA	2.01	1.95
Mean age at diagnosis (years)	13	21.9 ± 6.1	13.6	18	NA	16	15.1
TSS	177/208	13/14	12/13	1/8	12/13	4/6	219/262 (83%)
Biochemical control after first surgery	27/177	3/13	3/12	0/1	7/13	0/4	40/219 (18%)
Surgical reintervention	113/177	2/13	4/13	NA	1/7	2/6	122/219^a^
Biochemical control after second surgery	8/113	1/2	3/4	NA	NA	0/2	12/122^a^
SSA	118/208	0/14	6/12	8/8	3/13	5/6	140/262 (53%)
SSA biochemical control	0/118	0/14	1/6	0/8	NA	0/6	1/140^a^
PEG	37/208	0/14	2/6	4/8	1/13	2/6	46/262 (17.5%)
IGF-1 normalization with PEG	19/37	0	2/2	4/4	0/1	2/2	27/46 (58%)
RT	63/208	5/14	6/13	1/8	1/13	4/6	80/262 (30%)
Response to RT	27/63	3/5	NA	NA	1/1	2/4	33/80^a^
AIP mutation	42/208	NA	NA	3/8	5/13	2/6	52

AIP: aryl hydrocarbon receptor-interacting protein, NA: not available, PEG: Pegvisomant, RT: radiotherapy, SSA: somatostatin analogues, TSS: transsphenoidal surgery. ^a^ Percentage not reported due to incomplete data.

### Familial isolated pituitary adenomas

The diagnosis of FIPA should be suspected when 2 or more relatives have pituitary adenomas in the absence of known genetic causes, such as multiple endocrine neoplasia type 1, Carney complex, or McCune-Albright syndrome (22). The main causes of FIPA are X-LAG and *AIP* gene mutations.

X-LAG, which may occur isolated or associated with FIPA, is characterized by pituitary adenomas or pituitary hyperplasia producing most frequently increased levels of GH, GH releasing hormone (GHRH), and prolactin. The patients affected by this condition exhibit rapid growth starting in childhood. X-LAG is more frequent in women and responds poorly to treatment with SSAs (2,23,24).

Regarding mutations in the *AIP* gene, patients with these mutations inactivating the *AIP* gene generally have adenomas that produce GH and/or prolactin. Mutations of the *AIP* gene were reported in 5 out of 13 patients (38%) by Nagata and cols*.* (14), in 3 out of 8 (38%) patients by Mangupli and cols*.* (6), and in 42 out of 208 patients (20%) by Rostomyan and cols*.* (3). These mutations are characterized by early onset (before the age of 20 years) and frequent occurrence of gigantism, and for affecting males more frequently than females. Most tumors (93%) are macroadenomas and, compared with patients without *AIP* mutation, have a more aggressive behavior including greater extrasellar growth and lower response to surgical and medical treatment requiring a subsequent operation, and more frequent use of radiotherapy (2,25,26). This was evident in one of our patients (patient #1), whom even after two surgeries showed an increase in tumor size and absence of response to SSA, but finally responded to pegvisomant and radiotherapy. For these reasons, early screening of relatives of affected patients is important (26-28). Multiple mutations of this gene have been described. The heterozygous pathogenic variant of the *AIP* gene c.504G>A in exon 4 (p.Trp168*), found in our patients, has not been previously reported in the literature or in other patients in Colombia.

In our case series, sequencing of the *AIP* gene was requested from patients #1 and #5, taking into account the association of mutations of this gene with FIPA. Gene sequencing was also requested from all other patients, given the evidence of mutations of the *AIP* gene in children under 18 years of age with pituitary adenomas, and in those under 30 years of age with macroadenomas (29,30). However, this test was not approved by the health insurance of patients #2 and #3. Authorization for the test in patients #4 and #6 was pending at the time of the study, but these patients did not follow up at our Unit.

In conclusion, this is the largest case series described to date in Colombia of patients with gigantism, a pathology with a high functional and psychological impact on affected patients. Like other case series, men were more affected than women. It is important to note that the diagnosis was established late (at the age of 16 years) in our population compared with other studies. TSS was the first-choice procedure, but given a low biochemical control rate, pharmacological therapy was often required. It should be noted that the use of SSAs is less effective in gigantism than acromegaly, and that there are no significant differences in effectiveness between available analogues. In case of lack of response to SSAs, the association of pegvisomant is recommended. Even with an adequate biochemical response and symptom improvement, appropriate monitoring with tests should be performed due to the risk of tumor growth. The use of cabergoline (in patients with associated hyperprolactinemia) and radiotherapy as third-line management should be considered, but the high probability of radiotherapy-induced hypopituitarism in the pediatric population should be taken into consideration. To avoid continued vertical growth in patients with gigantism in cases of residual tumor and no response to surgery and SSA management, we consider that the best option in case of residual tumors is combined therapy with SSAs and pegvisomant. Pegvisomant as monotherapy can be considered in the absence of residual tumor, as well as in patients with the *AIP* gene mutation, given the high probability of therapeutic failure of SSA.

Mutations of the *AIP* gene should be considered in familial cases of GH-producing adenomas. Multiple pathogenic variants of this gene have been described, but this is the first time that these mutations have been documented in Colombia.
